# Analysis of the Mechanical and Preforming Behaviors of Carbon-Kevlar Hybrid Woven Reinforcement

**DOI:** 10.3390/polym13234088

**Published:** 2021-11-24

**Authors:** Zhengtao Qu, Sasa Gao, Yunjie Zhang, Junhong Jia

**Affiliations:** 1 College of Mechanical & Electrical Engineering, Shaanxi University of Science & Technology, Xi’an 710021, China; 1905014@sust.edu.cn (Z.Q.); 200511032@sust.edu.cn (Y.Z.); jhjia@sust.edu.cn (J.J.); 2 State Key Laboratory of Applied Optics, Changchun Institute of Optics, Fine Mechanics and Physics, Chinese Academy of Sciences, Changchun 130033, China

**Keywords:** hybrid woven reinforcement, preforming, mechanical properties, carbon-Kevlar

## Abstract

Carbon-Kevlar hybrid reinforcement is increasingly used in the domains that have both strength and anti-impact requirements. However, the research on the preforming behaviors of hybrid reinforcement is very limited. This paper aims to investigate the mechanical and preforming behaviors of carbon-Kevlar hybrid reinforcement. The results show that carbon-Kevlar hybrid woven reinforcement presents a unique “double-peak” tensile behavior, which is significantly different from that of single fiber type reinforcement, and the in-plane shear deformation demonstrates its large in-plane shear deformability. Both the tensile and in-plane shear behaviors present insensitivity to loading rate. In the preforming process, yarn slippage and out-of-plane yarn buckling are the two primary types of defects. Locations of these defects are closely related to the punch shape and the initial yarn direction. These defects cannot be alleviated or removed by just increasing the blank holder pressure. In the multi-layer preforming, the compaction between the plies and the friction between yarns simultaneously affect the quality of final preforms. The defect location of multi-layer preforms is the same as that of single-layer, while its defect range is much wider. The results found in this paper could provide useful guidance for the engineering application and preforming modeling of hybrid woven reinforcement.

## 1. Introduction

In recent years, the demand for excellent performance and lightweight materials has prompted the application of fiber-reinforced composite materials by replacing traditional metal materials [1]. The properties of reinforcements play an important role in composites. By controlling the orientation and volume fraction of fibers, composites can achieve the desired dimensional stability and mechanical strength while being formed into complex geometrical components [2,3,4,5,6]. Another approach to obtain the desired or improved performance is through hybridization among different types of reinforcements. Compared with traditional composites (single reinforcement type), hybrid composites have special properties and can meet a variety of design requirements in a more cost-effective manner. Some research has reported that there are many situations in which a high modulus material is needed, for example, carbon fiber reinforcements, but it is usually associated with a catastrophic brittle failure, which is not desirable [7]. The solution to such weakness is that the combination of carbon fibers and other types of fiber that have suitable ductility. For instance, carbon-Kevlar hybrid composite, which has excellent impact resistance, is a typical material used in military ballistic protection [8,9,10].

In the manufacturing of small and medium-sized composite parts, resin transfer molding (RTM) is the most common process. RTM requires the preforming of dry woven reinforcement and subsequent injection of resin. In this process, the reinforcement undergoes a change process from a 2D plane to a 3D geometric shape. The variations in the reinforcement, such as fiber volume fraction, deformation state, and other parameters, will have a great effect on the resin flow impregnation. In addition, the preforming defects observed at this stage, such as yarn slippage, buckling, and wrinkling, are closely related to the preforming behaviors [11,12,13,14]. The deformation behavior of reinforcement to form a specific shape primarily includes the deformation modes such as tension, in-plane shear, bending, and transverse compression [15,16,17], even the tension-shear coupling [18,19]. The deformation state of reinforcements strongly affects the mechanical properties of the final composite components [20,21,22]. Moreover, the reinforcement type (weaving type, number of layers, fiber orientation, etc.) and process parameters (such as punch shape, forming speed, pressure of the blank holder, etc.) can have a significant influence on the preform quality [23]. Therefore, in order to manufacture complex geometric composite parts without defects, it is important to understand the deformation behaviors during the preforming process. Numerical simulation allows researchers to adopt more effective and cost-effective design methods to study the feasibility of fabric forming. The simulation code describes the evolution of the behavior of the reinforcement through a mechanical method [14,19,24,25,26,27]. However, the accuracy of these numerical works must be verified by experimental results. It is important to study these defects and their mechanism so as to reduce their appearance. Labanieh et al. [22] studied the yarn slippage mechanism in the hemispheric preforming with woven carbon fabric. Gatouillat et al. [28] researched the yarn slippage defect and proposed an analytical model to predict the slippage. Capelle et al. [29] investigated the out-of-plane buckling of flax woven fabric during preforming and proposed a new special blank holder, which could reduce and sometimes even eliminate the occurrence of buckling. Furthermore, Shanwan et al. [30] developed a new strategy to understand the mechanism of mesoscopic defects in the preforming of woven fabrics and analyzed the effects of these defects on the mechanical properties of composites.

In practice, composite components are usually composed of multi-layer reinforcements, which can be arranged in different ply orientations to optimize the structural performance of the components. However, the deformation properties of multi-layer reinforcements have a close relationship with the relative orientation of plies. In this way, the occurrence possibility of defects is higher for multi-layer structures, especially for those with different ply orientations [31,32,33]. Moreover, the inter-ply friction prevents the relative sliding of plies, resulting in more severe wrinkles [31,34,35]. Guzman-Maldonado et al. [32] performed a numerical study on multi-layer preforming reinforcements. They investigated the interaction between adjacent plies during the preforming process and emphasized that the distortion of contact plies in different directions increased the severity of wrinkles. In addition, Thompson et al. [35] have proved that the stacking orientations can result in their own wrinkle pattern in the multi-layer preforming. Each ply became wrinkled due to the combined effect of its own intra-ply mechanical deformations and interactions with adjacent plies. Huang et al. [36] studied the effect of ply orientations on wrinkles of multi-layer reinforcement during bending. Severe wrinkling and interlaminar separation were observed in the alternate 0°/45° plies, while no wrinkling was observed in the pure 45° plies. Furthermore, Allaoui et al. [31] compared the preforming results of single-layer and multi-layer reinforcements by quantifying the shear angle and defects. Preforming defects of multi-layer reinforcements occurred in the same location as those of single-layer reinforcements but in a wider range.

From the above literature, it can be found that much research has been conducted on the preforming behavior of woven reinforcement fabrics. However, most of them are for the single fiber type reinforcement, while few reports are for the hybrid reinforcement. As a promising composite material, the performance of carbon-Kevlar hybrid woven reinforcement may be different from that of pure carbon fiber reinforcement or pure Kevlar fiber reinforcement due to the interaction of the two kinds of fibers. There exists some research on the carbon-Kevlar hybrid composites [8,37,38], indicating that appropriate fiber hybridization could improve the mechanical performance of composites. However, these studies are primarily focused on the cured composites composed of carbon-Kevlar hybrid reinforcements, and the knowledge of the mechanical properties of dry hybrid reinforcements is far not sufficient. Due to the brittleness of carbon fibers, the fracture strain of carbon fibers (about 1%) is much lower than that of Kevlar fibers (about 3.5%), which makes them more vulnerable to damage during stretching and even preforming processes.

The objective of this work is to investigate the mechanical properties of carbon-Kevlar hybrid woven reinforcements and their possibilities to form complex shapes. A main concern is the defects that occur during the preforming process. Several different preforming process parameters are designed to investigate the mode of defects under specific conditions. The results and defects of single-layer and multi-layer preforming are compared quantitatively.

## 2. Materials and Methods

### 2.1. Tested Materials

**Figure 1:**
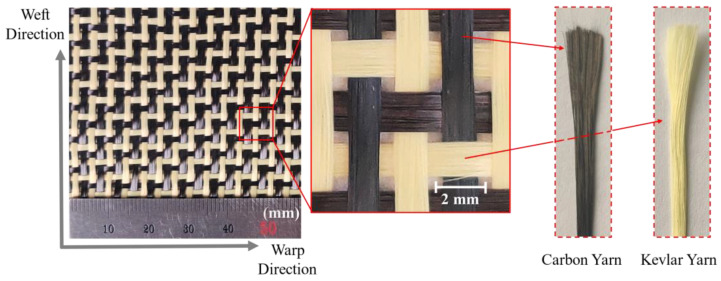
Tested carbon-Kevlar hybrid woven reinforcement.

The carbon-Kevlar hybrid woven reinforcement (Shanghai Banglin Composite Material Technology Co., Ltd., Shanghai, China) used in this study is shown in Figure 1, which consists of two yarn networks in two directions (warp and weft), with carbon fiber yarn and Kevlar fiber yarn in each direction. The reinforcement possesses a 1/3 twill woven structure. Compared with plain woven structure, it has a reduced crimp level and better drapability, while the interlocking between warp and weft yarns is not that tight. These factors result in high permeability and better suitability for the RTM process. The main material parameters of carbon-Kevlar hybrid woven reinforcement are shown in Table 1.

### 2.2. Mechanical Behavior Characterizations

**Figure 2:**
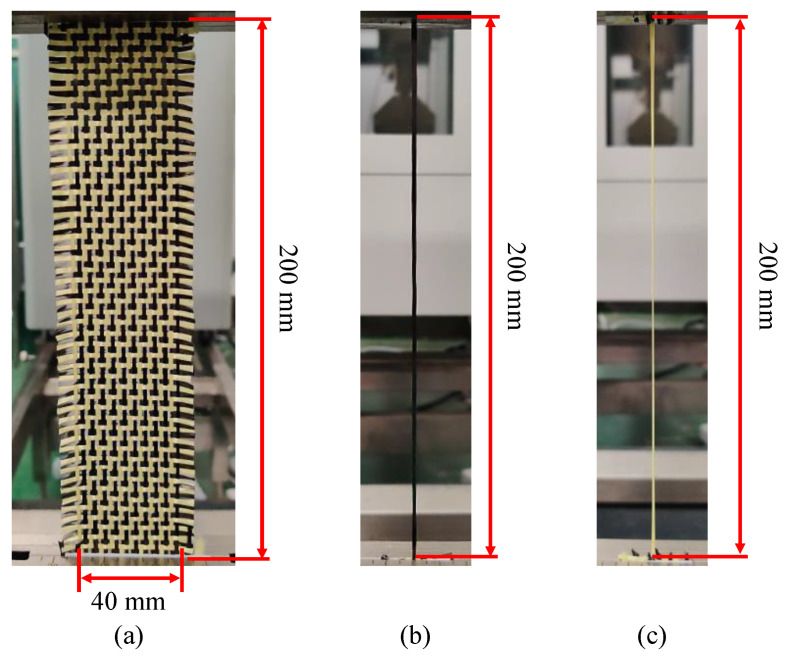
Tested specimen in the tensile test: (**a**) hybrid woven reinforcement; (**b**) carbon yarn; (**c**) Kevlar yarn.

There are complex relationships among mechanical properties of reinforcements, such as preforming process parameters and punch shapes, which affect the quality of final preforms. Firstly, the mechanical characterization of the hybrid woven reinforcement was investigated. The interested deformation behaviors of hybrid woven reinforcements are the tensile deformation along yarn direction and in-plane shear between two yarn directions, which are the primary deformation mode in preforming. In the tensile tests, it can be noted that the deformation behavior of hybrid woven reinforcement was significantly different from that of a single fiber type of reinforcement. Although yarns will not be broken in the preforming process, the tensile fracture behavior of carbon-Kevlar hybrid woven reinforcements was still analyzed considering their actual applications (usually used in the field of protection). The phenomenon was interesting because it visualizes the advantages of hybridization. The tensile test conducted in this study included two aspects: one was for the hybrid woven reinforcement, and the other was for single carbon yarn and single Kevlar yarn in order to better explain the failure mechanism of hybrid woven reinforcement. The selected geometry of specimens in the tensile test is sketched in Figure 2. The effective area of specimens (200 × 40 mm^2^) contains 10 carbon yarns and 10 Kevlar yarns in the longitudinal direction (Figure 2a). Different loading speeds (2, 10, 50, 10, 200, and 400 mm/min) were applied to investigate the sensitivity of loading speed on the hybrid woven reinforcement. Moreover, in order to analyze the contribution of different kinds of yarns to the global mechanical behaviors of hybrid woven reinforcement, the tensile behavior of single carbon yarn and single Kevlar yarn extracted randomly from raw material were tested. For yarn samples, the gauge length was 200 mm (Figure 2b,c), and the loading speed was set as 10 mm/min. In addition, the deformation and failure behaviors of the specimens were recorded by a high-resolution camera at the sampling rate of 2 Hz. Each of the specimen configurations was tested at least three times to assess repeatability.

**Figure 3:**
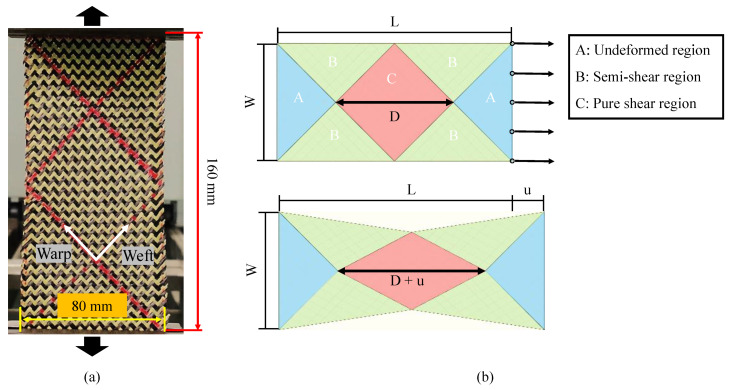
Tested specimen in the bias-extension test: (**a**) tested specimen; (**b**) illustration of the specimen before and after deformation.

The in-plane shear is the primary deformation mode in the preforming of double-curved shape. Physical phenomena such as contact and friction between yarns are related to shear properties. During the RTM process, the in-plane shear deformation could cause a significant change in the permeability of reinforcement, especially when the shear angle reaches the “locking angle”. Although there is no direct relationship between shear angle and wrinkling, an excessive shear angle is more likely to cause wrinkling [16]. In this study, the in-plane shear properties of hybrid woven reinforcement were investigated by the bias-extension test. Compared with the picture frame test, the bias-extension test is relatively simple and can be performed on a tension machine without other special devices. The specimen was a rectangular piece with an angle of ±45° between the yarn orientations and the loading direction (Figure 3), whose length (L = 160 mm) is at least twice as long as the width (W = 80 mm). Red marker lines have been drawn on the specimens, which would be used for tracking shear angle evolution. To rule out the influence of the yarn slippage on the shear angle that is directly determined from the displacement, a high-resolution camera was used to take the images. The shear angle can then be accurately measured with ImageJ software to process images taken by the camera. In the current work, the bias-extension tests were conducted under different loading speeds of 2, 10, 50, and 200 mm/min.

### 2.3. Preforming Tests

**Figure 4:**
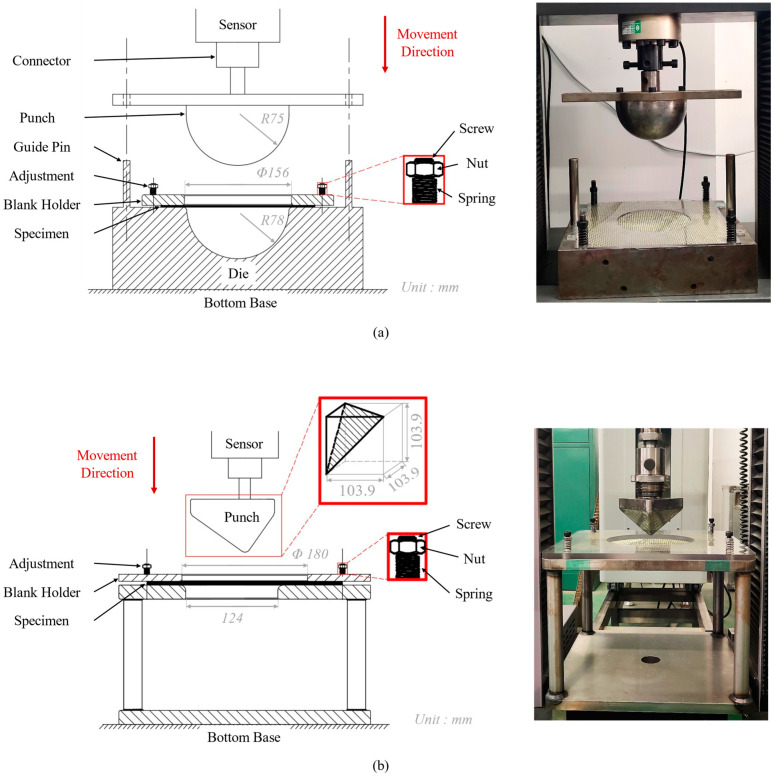
The hemispherical and tetrahedral preforming device: (**a**) experimental set-up for hemispherical preforming; (**b**) experimental set-up for tetrahedral preforming.

The carbon-Kevlar hybrid woven reinforcement is usually used in cases with both strength and anti-impact requirements, such as a protective helmet or car bumper, which usually includes complex double-curved shapes. Considering its special applications, classic hemispherical and tetrahedral punches were used to investigate the feasibility of hybrid woven reinforcements to form specific shapes. The punch was installed on the universal testing machine (Shenzhen Wance Testing Machine Co., Ltd., Shenzhen, China) to realize the preforming test. The movement of the punch was controlled and measured by an electric jack equipped with a sensor, which also can measure the position and the loading speed of the punch. The first punch was a hemisphere with a diameter of 150 mm, and the punch stroke was 75 mm (Figure 4a). The gap between the punch and die was 3 mm. The other open tetrahedral die is shown in Figure 4b. The shape of the tetrahedral punch is the part cut on a cube with an edge length of 103.9 mm and a radius of 7.5 mm at its edge corner. The gap between the punch and die was 1.5 mm, and the punch stroke was 60 mm. The adjustment was installed in the four corners of the blank holder in both dies, which can achieve variation blank holder pressures by adjusting the spring compression. Additionally, the spring stiffness related to the holder pressure should be measured by independent experiments before preforming tests.

**Figure 5:**
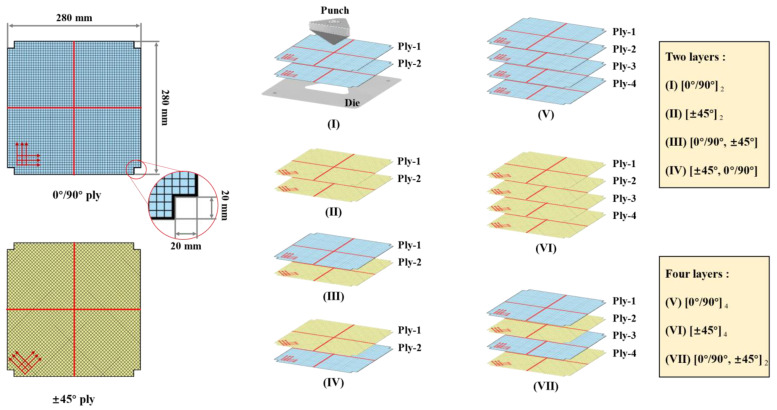
Schematic of specimen size (suitable for hemisphere preforming and tetrahedral preforming) and multi-layer configurations (only for tetrahedral preforming).

The specimens used in the experiment of hemispherical preforming and tetrahedral preforming are same (280 × 280 mm^2^). The rectangular with a side length of 20 mm was cut from the four corners of the specimen to prevent interference with the adjustments (Figure 5). For woven structures, it is essential to study the effect of initial yarn orientation (such as 0°/90° and ±45°) on the preforming properties [39], especially for forming complex shapes. Therefore, two initial yarn orientations (0°/90° and ±45°) will be considered in this study. Additionally, the quality of the final preform is also affected by the tension force imposed on the specimen during the preforming process, which can cause other defects [40,41]. Therefore, several low (0.08 Kpa for hemispherical preforming and 0.25 Kpa for tetrahedral preforming) and high (3.7 Kpa for hemispherical preforming and 4 Kpa for tetrahedral preforming) blank holder pressures were chosen for single-layer preforming experiments in this study. After preforming, the deformed reinforcement needs to be cured with the structural glue to avoid spring-back after removing the blank holder. This structural glue contains acrylic resin and a curing agent. After the two are mixed, they can be quickly cured at room temperature (usually only a few minutes). The specimen can be removed from the die after the curing of the glue for further measurement.

After the preforming process, yarn orientation and shear angle distribution are particularly essential to the permeability of the reinforcement and the mechanical behavior of the final composite product. When the preform was multi-layered, especially for that with different initial ply orientations, the occurrence possibility of defects would increase greatly [32]. In tetrahedral preforming, significant strains and additional defects occurred in the specimen due to the low punch edge radius. Therefore, tetrahedron preforming experiments have been carried out to analyze the interaction between different plies and their effect on the defects. These experiments were performed with low blank holder pressure (0.25 Kpa) and a punch loading speed of 10 mm/min. The influence of the ply orientation was also considered, and the configurations of multi-layer preforming were shown in Figure 5. The center position of the sample was marked with red marker lines for positioning. The two-layer preforms were fixed with resin after preforming. However, for four-layer preforms, it was tricky to observe the deformation of the inner layer due to the solidification. Therefore, it was necessary to carefully remove the upper layer, and the remaining ply was measured and analyzed after each removal. Then, the preforming results of different configurations were compared in order to analyze the influence of different ply orientations on the multi-layer preforming.

## 3. Results and Discussion

### 3.1. Mechanical Behavior Characterization

#### 3.1.1. Tensile Behaviors

**Figure 6:**
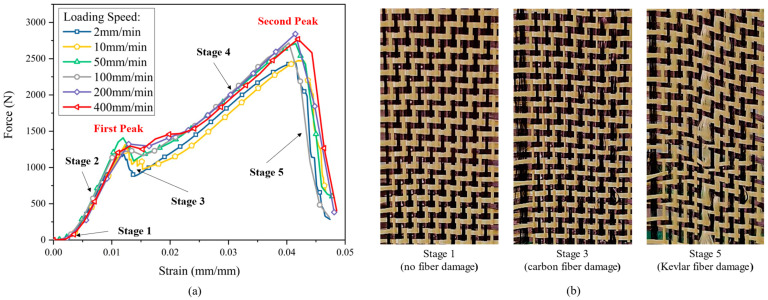
Tensile test results of hybrid woven reinforcement: (**a**) the force-strain relationship under different loading speeds; (**b**) the deformation and damage behaviors in some typical stages.

Figure 6 presents the curves of the tensile force versus strain at different loading speeds. The characteristics of tensile behavior are significantly different from those of reinforcement with a single fiber type. In the curve, a unique “double-peak” feature was reflected, which was related to the different properties of the two kinds of yarns. The fracture of carbon fiber yarns was directly related to the formation of the first peak, and the subsequent behavior largely depended on Kevlar yarns.

The whole curve can be divided into five stages (Figure 6). In stage 1, since the wave-shaped yarn was straightened in the loading direction, the stress increase was relatively low. In stage 2, the tensile curve showed a linear behavior. Both carbon yarns and Kevlar yarns were straightened with the increase in strain. In stage 3, carbon yarns firstly came to be broken due to their small ultimate strain. The curve reached the first peak. Severe fluctuations in the curve can be seen during this stage, which is due to uncertain defects existing in certain carbon yarns, and the fracture of carbon yarns may not occur at the same time. In addition, the fracture of carbon yarn will lead to the redistribution of force in the remaining yarns and a stiffness reduction in the reinforcement. Thus, there has a force decreasing as shown in stage 3. In stage 4, with the strain increased continually, the Kevlar yarns were still undamaged and continued to be stretched. Finally, the curve reached the second peak. The Kevlar yarns began to be damaged after reaching their ultimate strain, which represented the complete failure of the whole material, as shown in stage 5.

**Figure 7:**
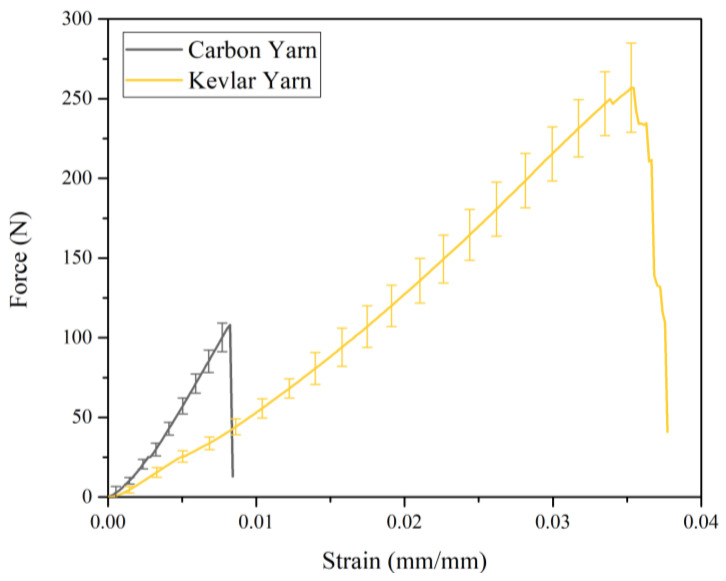
Tensile force versus strain curves of carbon yarn and Kevlar yarn at a loading speed of 10 mm/min.

**Figure 8:**
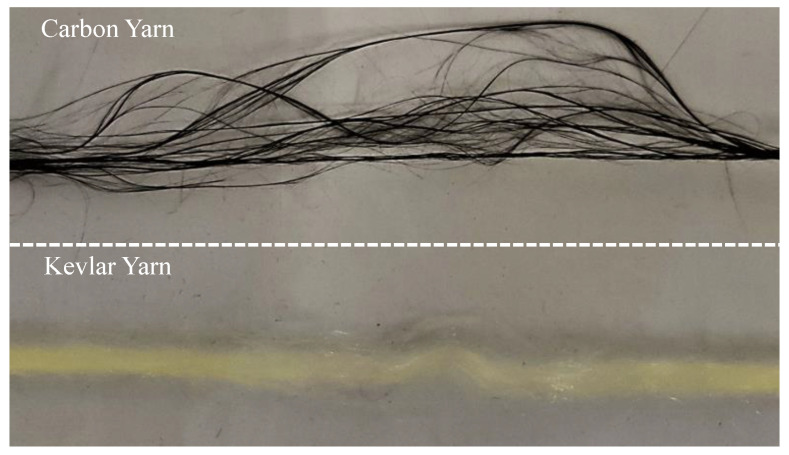
Failure modes of two different yarns.

The tensile behavior of carbon-Kevlar hybrid woven reinforcement can be further elucidated by that of single carbon yarn and single Kevlar yarn. The tensile force-strain curves of both carbon yarn and Kevlar yarn are shown in Figure 7. It is worth noting that stage 1 in Figure 6 was not presented in Figure 7 since the yarn was straight at the beginning. As can be seen in Figure 7, carbon fiber provides better initial tensile stiffness, while Kevlar fiber provides better tensile strength for the hybrid woven reinforcement. Therefore, hybrid woven reinforcement materials have excellent energy absorption properties and can be applied on occasions with anti-impact requirements. In addition, the carbon yarn and the Kevlar yarn exhibited different failure modes, which can be seen in Figure 8. The fracture of carbon yarn appeared an obvious brittleness, and the energy was released instantly when the yarn was broken and then exploded outward. In contrast, the Kevlar yarn had larger toughness and formed a fluffy shape after breaking.

#### 3.1.2. In-Plane Shear Behaviors

**Figure 9:**
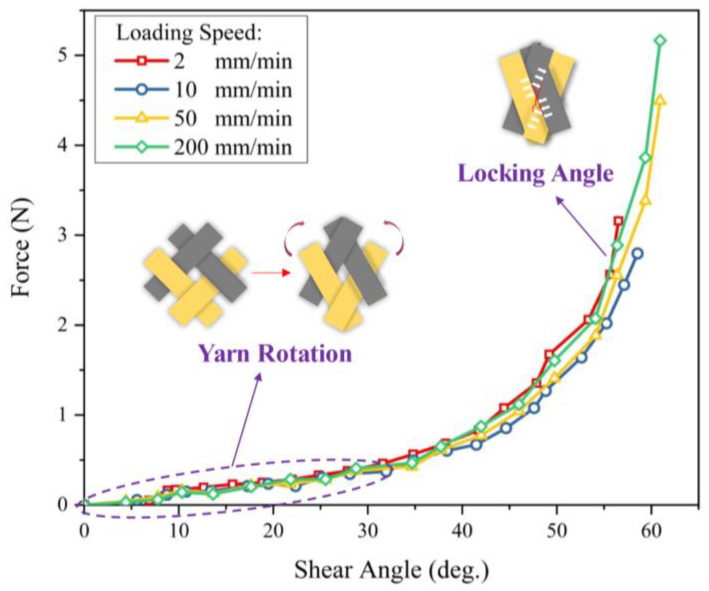
Force-shear angle curves at different loading speeds in bias-extension test.

**Figure 10:**
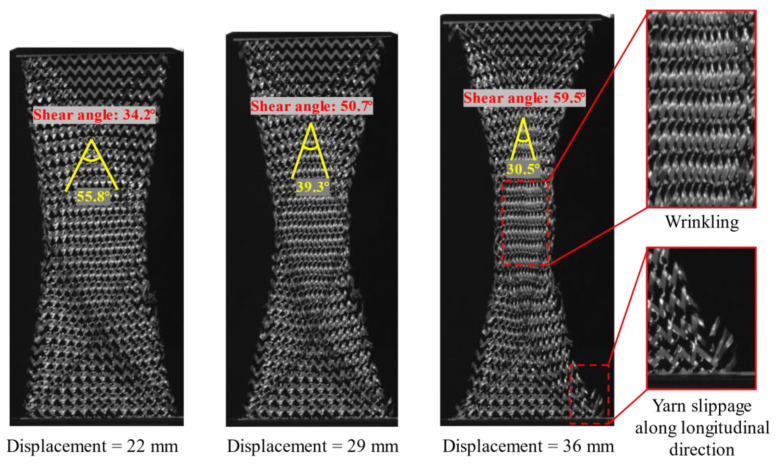
Shear angle evolution in the bias-extension test. The wrinkles occur at a shear angle of about 60°.

Figure 9 presents the curves of the clamp force versus shear angle. It is clearly indicated that the shear behavior was also insensitive to the loading speed. Two obvious deformation phenomena can be noted. At the initial stage, the force resistance primarily comes from the friction between yarns, and yarn free rotation can be seen. As the rotation continues, the gap between adjacent yarns decreases and yarns begin to contact and compress with each other, and the load increases significantly with the increase in shear angle. Significant yarn slippage along the longitudinal direction occurred during the bias extension due to the very loose weaving structure. Furthermore, the slippage occurred at four corners of the specimen due to the yarns at this position being subjected to the least friction. Figure 10 shows the evolution of shear angles under different displacements, and three different zones corresponding to the theoretical kinematics of the test (Figure 3b) were clearly observed in the deformed specimen. Additionally, the shear stiffness of the reinforcement was low, with slight wrinkles occurring at a shear angle of about 60°. The experimental results indicate that the reinforcement used in this study can adapt to large shear deformation with high locking angle (55°~60°). Thus, the hybrid reinforcement has suitable formability. Nevertheless, yarn slippage is a major concern, especially in the process of shaping a deep-draw shape.

### 3.2. Preforming of Hybrid Woven Reinforcement

Since the reinforcement rarely breaks during the RTM processing, the double-peak tensile properties have little effect on the preforming behavior of hybrid woven reinforcements; generally, only the deformation stage before the damage of carbon yarn needs to be considered. The in-plane shear deformation is one of the main deformation modes of reinforcement in the preforming process. From the bias-extension test, it is known that yarn slippage is inclined to occur since the weaving structure of the carbon-Kevlar reinforcement is relatively loose. Furthermore, the friction behaviors in multi-layer preforming are more complex because of the relative motion between plies, which can lead to the possible enlargement of defect occurrence. In this section, we will analyze and discuss the key factors that affect the quality of preform when preforming with single-layer and multi-layers.

#### 3.2.1. Single-Layer Preforming

##### Deformation of Preforms with Different Initial Yarn Directions

**Figure 11:**
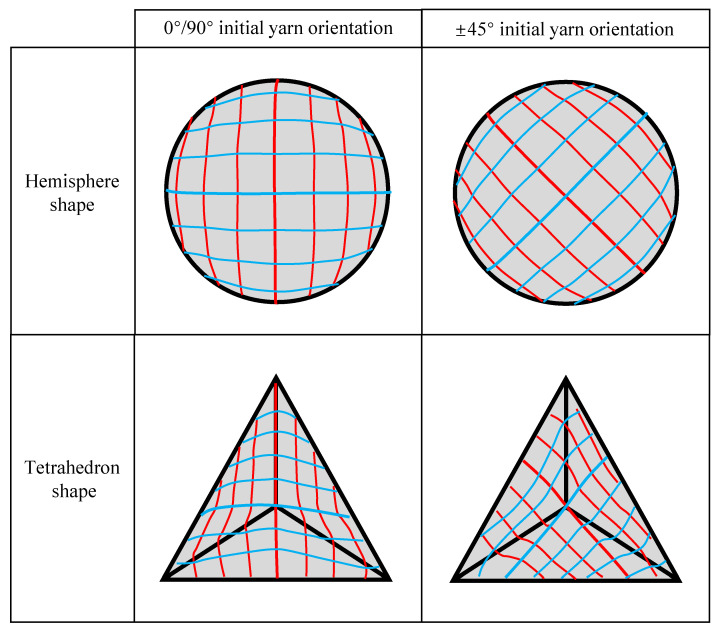
Yarn orientations after preforming for the hemispherical and tetrahedral preforms. Red lines for weft yarns; blue lines for warp yarns.

**Figure 12:**
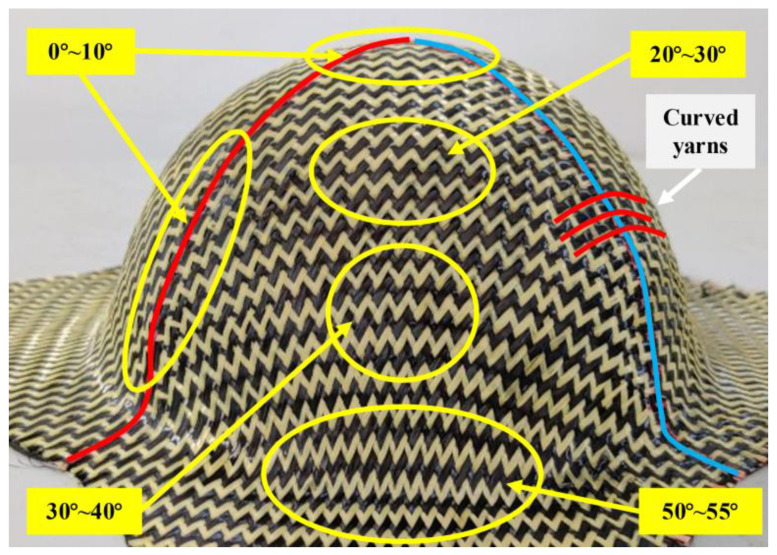
Shear angle distribution after hemispherical preforming. Red lines for weft yarns; blue lines for warp yarns; yellow lines for shear zones.

The feasibility of hybrid woven reinforcement forming into complex curved parts with two different initial yarn orientations was studied by using hemispherical and tetrahedral punches, respectively. The yarn orientation after preforming was analyzed firstly since it directly affects the mechanical properties of the final part and the permeability of the preforming. In the experiment, preforms with suitable shape quality were obtained at a punch loading speed of 10 mm/min and a low blank holder pressure (of 0.08 Kpa for hemispherical preforming and 0.25 Kpa for tetrahedral preforming). Figure 11 schematically depicts the final yarn orientation in the useful area (i.e., the area not covered by the blank holder) after preforming. Due to the symmetry of the hemisphere punch, there is almost no difference for the material draw-in for the preforms with different initial yarn orientations, indicating that the initial yarn orientation did not affect the quality of hemispherical preforms. Furthermore, the shear angle distribution of hemispherical preform in the useful area is shown in Figure 12. The shear angle varied continuously, and clearly distinguished shear zones cannot be observed. The area near the blank holder had a higher shear angle due to larger material draw-in, and the maximum shear angle can reach 55°. However, no obvious wrinkling was observed. In addition, yarns usually undergo in-plane orientation change at the intersectional position with the central warp and weft yarn, resulting in a noticeable curve, shown as a red line in Figure 12, and near which the shear angle was usually small (close to 0°).

**Figure 13:**
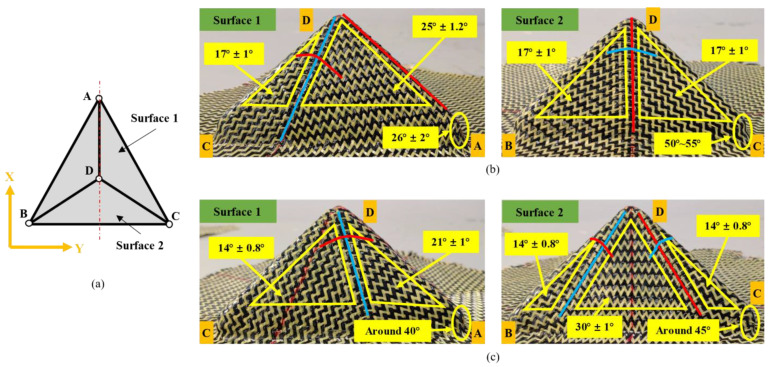
Shear angle distribution after tetrahedral preforming: (**a**) schematic diagram of tetrahedral preform description; (**b**) surface 1 (left) and surface 2 (right) of the tetrahedral preform with 0°/90° initial yarn orientation; (**c**) surface 1 (left) and surface 2 (right) of the tetrahedral preform with ±45° initial yarn orientation. Red lines for weft yarns; blue lines for warp yarns; yellow lines for shear zones.

However, for the tetrahedron, the initial yarn orientation has a great influence on the behavior of the preform, and the shear deformation was more complex. In addition, changes in yarn orientation also occurred at the intersection edges of different planes. As can be seen in Figure 11, the preform with ±45° initial yarn orientation underwent more yarn orientation changes. Furthermore, Figure 13 gives the shape and shear angle distribution of the tetrahedral preforms with different initial yarn orientations. Unlike hemispheric preforms, clearly distinguished shear zones could be observed in tetrahedral preforms. The dividing line between these shear zones was usually the central warp and weft yarn. It also existed the characteristic that the shear angle at the central warp and weft yarn position was close to 0°. The yarns in the upper triangular pyramid section of the preform have a suitable alignment structure, but the shear angle becomes bigger at the bottom of the preform, especially at the edge with small curvature. The maximum shear angle can achieve 50°~55° in the preform with 0°/90° initial yarn orientation, which is a little bigger than that of ±45° initial yarn orientation. Similarly, with the hemispherical preform, no wrinkles were observed in tetrahedral preforming with different initial yarn orientations. The main reason was that the loosely weaving structure prevents wrinkling even under high shear deformation. In addition, the yarn experienced a large orientation variation at the intersection of central warp and weft while the shear angle in this region was close to 0°.

**Figure 14:**
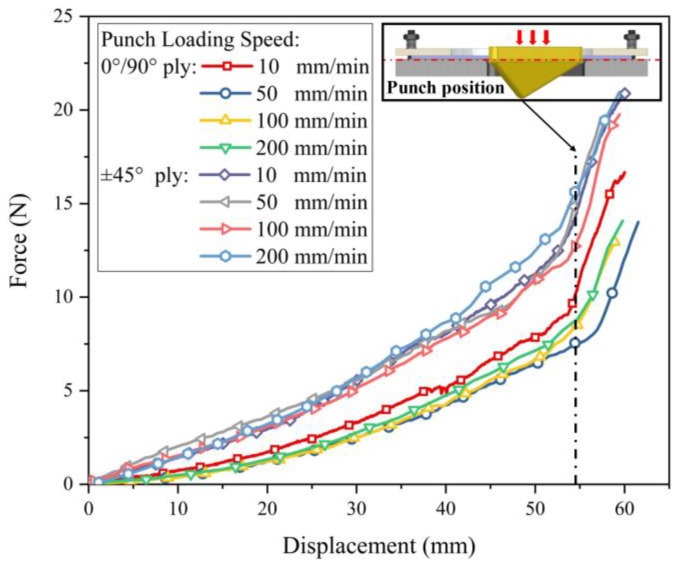
Punch force of reinforcements with 0°/90° and ±45° initial yarn orientations at different forming speeds.

Figure 14 gives the punch force of reinforcements in 0°/90° and ±45° initial yarn orientations at different forming speeds. For hybrid reinforcement with the same initial yarn orientation, the punch loading speed had little effect on the punch force. The deformation modes of the reinforcement were similar under constant blank holder pressure with different punch loading speeds. However, the punching force of the reinforcement in the ±45° initial yarn orientation was larger. Since the punching force is to overcome sliding friction and shear forces, it means that the reinforcement in the ±45° initial yarn orientation underwent larger sliding or shear deformation. In general, the effect of initial yarn orientation is on the punch shape. For hemisphere preforming, the results show that the initial yarn orientation has little effect on the preforming. However, when it comes to the more complex tetrahedral shape, the initial yarn orientation has a great influence on the quality of the preform. Variations of the initial yarn orientation may not cause significant defects but will result in changes in final yarn orientation [41], which directly affect the local permeability of the reinforcement and the mechanical properties of the composite parts.

##### Preforming Defect Description of Hybrid Woven Reinforcement

**Figure 15:**
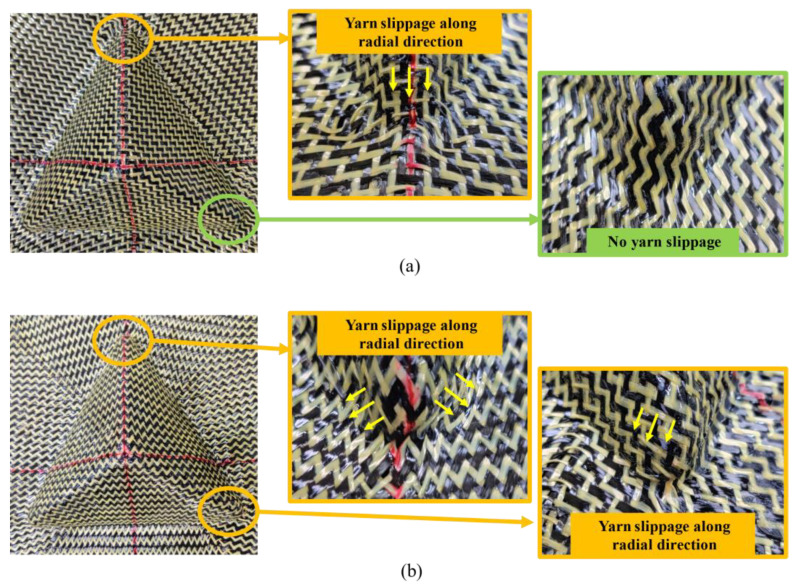
Yarn slippage along radial direction occurred at the bottom corner of the tetrahedral preform: (**a**) tetrahedral preform with 0°/90° initial yarn orientation; (**b**) tetrahedral preform with ±45° initial yarn orientation.

During the preforming process of single-layer hybrid woven reinforcement, there mainly exist two kinds of defects: yarn slippage and out-of-plane yarn buckling. For tetrahedral preforms, a typical defect is yarn slippage along the radial direction, occurring at the bottom corner of the preform, as shown in Figure 15. This random slippage made it possible to observe irregular gaps at the bottom corners of the preform and will have a strong influence on the local permeability of the preform. In single-layer preforms, such defects were located in non-useful areas, which would be cut off after forming process and not affect the quality of preform. However, this kind of defect should be taken into account when it comes to multi-layer preforming because the interaction between plies may magnify the defect. In addition, no yarn slippage was observed in the preform with an initial yarn direction of 0°/90° (Figure 15a, shown in the green zone), which indicated that yarn slippage can be reduced by properly adjusting the initial yarn direction.

**Figure 16:**
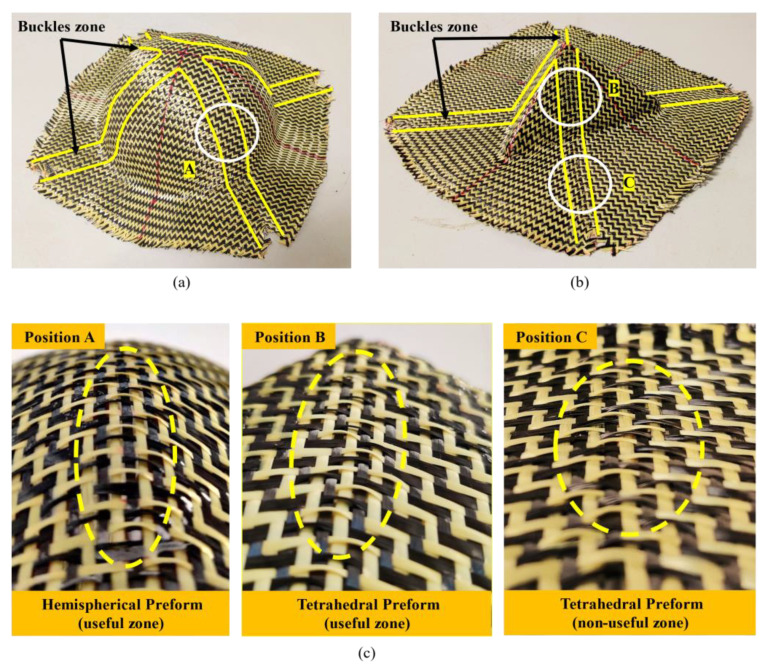
Out-of-plane yarn buckling in the preform and its location. Located near the central warp and weft yarn: (**a**) buckles zone on tetrahedral preform; (**b**) buckles zone on hemispherical preform; (**c**) zoom of the buckles.

Another kind of defect, out-of-plane yarn buckling, occurred in both tetrahedral and hemisphere preforms. Such defects converge from the bottom to the top of the preform, and their position depends on the initial yarn direction. To be exact, they were located near the center warp and weft yarn. Buckling is a yarn scale phenomenon that usually only involves individual yarns and does not result in any membrane strain [31]. As shown in Figure 16, the yarns, which were intersected with the central warp and weft yarn, were subject to out-of-plane yarn buckling since they were impossible to accommodate in-plane bending in a small area. The magnitude of yarn buckling depended on the curvature of the in-plane bending and was proportional to the in-plane bending. These local buckling would cause a change in the thickness of the material and also affect the permeability of the reinforcement because of the conservation of yarn volume. Buckling is an unacceptable defect; thus, appropriate forming process parameters should be studied to reduce the occurrence of such defects.

##### Influence of Blank Holder Pressure on Defects

**Figure 17:**
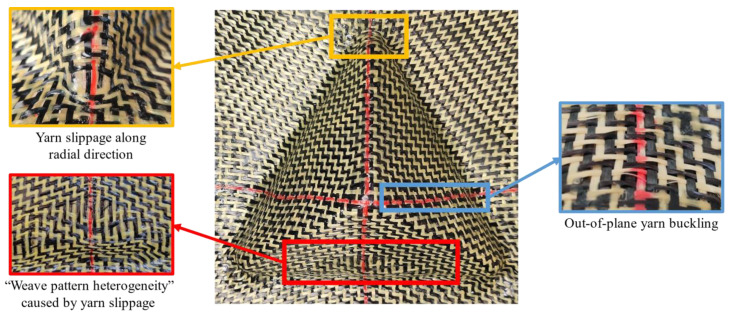
The preforms shape obtained from a tetrahedron preforming under high blank holder pressure (4 Kpa). Excessive yarn tension leads to more serious yarn slippage along the radial direction, but out-of-plane yarn buckling does not disappear.

The blank holder pressure plays a very important role in the composite-forming process [42]. The blank holder pressure imposes tension on the reinforcement, which is also called preloading. The increase in blank holder pressure tends to delay the appearance of wrinkles. This means that if tension is applied to the reinforcement and even the shear angle is higher than the “locking angle”, it is possible to obtain high shear deformation without wrinkles. For the carbon-Kevlar hybrid woven reinforcement used in this study, slight wrinkling could be observed when the shear angle reached 60° in the bias-extension test. However, wrinkles did not occur in the preforming. The influence of different blank holder pressure on the defects was quantified in this study. The preforms obtained by a tetrahedron preforming under high blank holder pressure (4 Kpa) are shown in Figure 17. The yarn slippage existed at the bottom corners of the preform, and the “weave pattern heterogeneity” phenomenon caused by yarn slippage along radial direction can be observed in the useful area. When the friction between the yarn/yarn and the yarn/punch is not enough to resist this tension, yarn slippage occurs. This type of defect is obviously unacceptable. In addition, out-of-plane yarn buckling would still exist when the pressure on the blank holder was large enough. Studies have shown that out-of-plane yarn buckling can be avoided by increasing the pressure on the blank holder [43]. However, for the carbon-Kevlar hybrid woven reinforcement in this study, this method did not seem to work since out-of-plane yarn buckling was noted even at high blank holder pressure (4 Kpa). In future work, related research includes optimizing the structure of the reinforcement should be carried out to reduce such defects.

**Figure 18:**
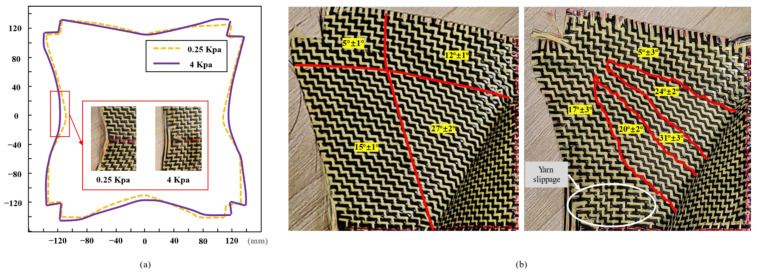
0°/90° tetrahedral preforms under different blank holder pressures. A decrease in material draw-in and an uncertain change in shear angle distribution occur at high pressure: (**a**) material draw-in comparison; (**b**) shear angle distribution of non-useful areas under 0.25 Kpa (left) and 0.4 Kpa (right) blank holder pressure.

Another interesting phenomenon that occurred in the non-useful area caused by blank holder pressure was also analyzed. As shown in Figure 18, in the tetrahedral preform with 0°/90° initial yarn orientation, less material draw-in under high blank holder pressure can be observed because the movement of the yarn was restricted by the pressure of the blank holder (Figure 18a). The preform obtained under low blank holder pressure had a suitable symmetrical profile while existing little deviations. One of the reasons was that it was difficult to ensure the specimen was placed on the mold with complete symmetry. On the contrary, the profile of the preform obtained under high blank holder pressure was more distorted, which indicated that the yarn movement was discontinuous. In addition, the randomness of shear angle distribution could also be observed in non-useful areas. Since the increase in shear angle can cause the local thickness changes, it makes the measurement of shear angle difficult. When the material thickness changes, the pressure exerted by the blank holder on the material is uniform, which may affect the friction coefficient between local materials and tools. This non-uniformity of friction coefficient can lead to different yarn slippages. Since the yarn cannot be extended, it can affect the shear angle in the useful area and even produce buckling defects. It can be seen that the influence of blank holder pressure on preform quality is very complex. In order to further understand this phenomenon, it is necessary to develop a specific test independent of the process. In actual production, this process is difficult, and corresponding optimization research can be carried out through numerical simulation on the mesoscopic scale [44].

#### 3.2.2. Multi-Layer Preforming

##### Defects in Multi-Layer Preforms with Different Stacking Configurations

In order to study the deformation behavior and possible defects of the multi-layer preforming hybrid woven reinforcements, tetrahedral preforming experiments with different ply configurations were carried out. The test was performed at low blank holder pressure (0.25 Kpa) and punch loading speed of 10 mm/min. The multi-layer configurations used in this study are shown in Figure 5, and the corresponding sequences are used to indicate the layering situation in the following description (for example, “Configuration I” represents the two plies are both 0°/90°, see Figure 5 for more details). In the multi-layer preforming, all the defect types observed in the previous single-layer preforms can also be seen and even more complex. Similarly, wrinkling can be completely suppressed under low blank holder pressure, as analyzed in single-layer preforming. However, there were several key locations worth noting where defects occurred in single-layer preforms (as mentioned in Section “Preforming Defect Description of Hybrid Woven Reinforcement”). When it comes to a multi-layer preforming, defects will be magnified due to the inter-ply friction. Although the yarn slippage occurred in non-useful areas in single-layer preforms, it should also be noted that the influence of these defects will not spread to useful areas in multi-layer preforms.

**Figure 19:**
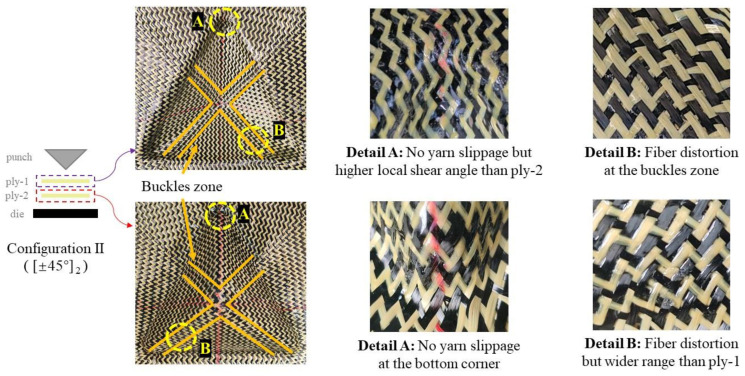
Defects in preform with the consistent orientation between plies.

Figure 19 shows the defects observed in the multi-layer preform (configuration II) with consistent relative orientation between plies. It can be observed that the preform had no yarn slippage at the bottom corner, which is due to the difficulty of yarn slip caused by inter-ply friction at this position. In addition, the local shear angle of the inner layer (ply-1) was larger than that of the outer layer (ply-2). The difference in radius was one of the possibilities that led to this phenomenon. Due to the volume conservation in the in-plane shear process, the thickness of the reinforcement increases [45]. In the multi-layer preform containing 0°/90° ply orientation, although the yarn slippage at the bottom corner did not disappear, they did not affect the useful area. Therefore, this defect can be ignored. However, the fiber distortion can be observed near the buckles zone. This phenomenon was induced by the influence of the interaction of the plies since the buckles zone between the plies were at the same location. In this position, the local volume fraction of the preform changed greatly, which would affect the mechanical properties. In addition, the range of fiber distortion in the inner layer (ply-1) was narrower than that in the outer layer (ply-2). This may be due to the compaction. The compaction provided by the outer layer can reduce some defects in the inner layer to a certain extent. However, this compaction did not cause the loss of buckling. In general, for multi-layer preforms with consistent relative orientation between plies, the increase in inter-ply friction caused by out-of-plane yarn buckling was the main influence on the occurrence of defects in multi-layer structures. In some cases, inter-ply friction is more important than compaction, especially when out-of-plane deformation occurs.

**Figure 20:**
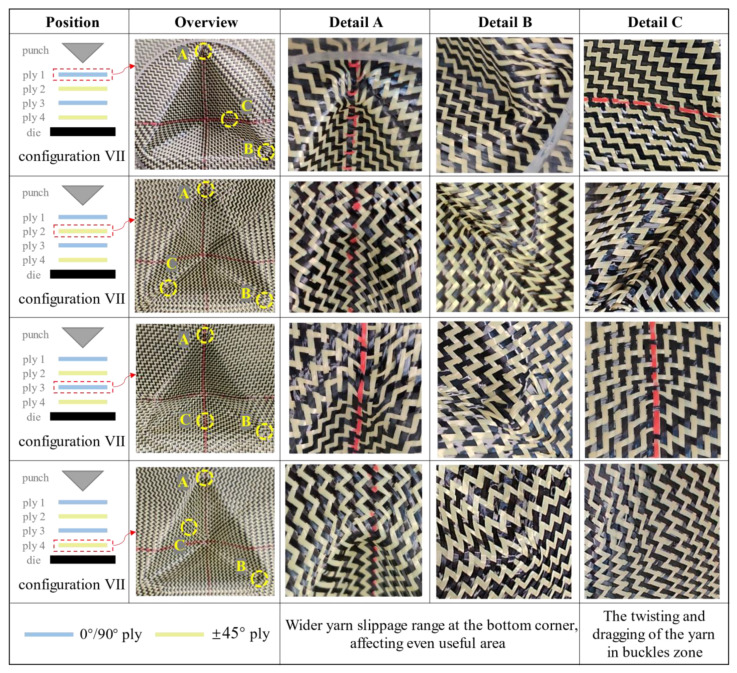
The deformation of each ply in configuration VII. Differences in relative deformation patterns between plies lead to a wider range of yarn slippage at the bottom corner, and yarn pull-out was observed in the buckles zone.

For multi-layer preforms with inconsistent relative orientation between plies, different deformation modes can lead to more serious defects. Figure 20 lists the deformation of each ply in configuration VII. Detail A and detail B correspond to the two bottom corners of the preform, which is the location where yarn slippage along radial direction was easy to occur. It can be seen that under the superposition of multi-layer defects, the deformation of the preform in this place was unsatisfactory, even affecting the useful area. Detail C corresponds to the location where buckling occurs in each ply. Although the buckling positions of adjacent plies were different, they would still affect each other. Under the influence of different deformation modes, the relative slippage between plies was more severe. It can be observed that the yarns were pulled out of the weaving structure at the buckles zone. From the whole preforming results, the location of defects produced by multi-layer preforms was the same as that of single-layer preforms while the range was larger. The factors causing defects were mainly attributed to the inter-ply friction. For the surface with only in-plane shear deformation, the friction is relatively small. The inter-ply friction will not affect the preforming quality, and even better deformation behavior is represented under the action of compaction. However, when out-of-plane deformation occurs, the friction would increase rapidly, which would directly affect the quality of preforms. Fully understanding these friction phenomena is a prerequisite condition for obtaining ideal preforms. Thus, the research on inter-ply friction needs to be further studied.

##### Inter-Ply Friction Mechanism in Multi-Layer Preforming

**Figure 21:**
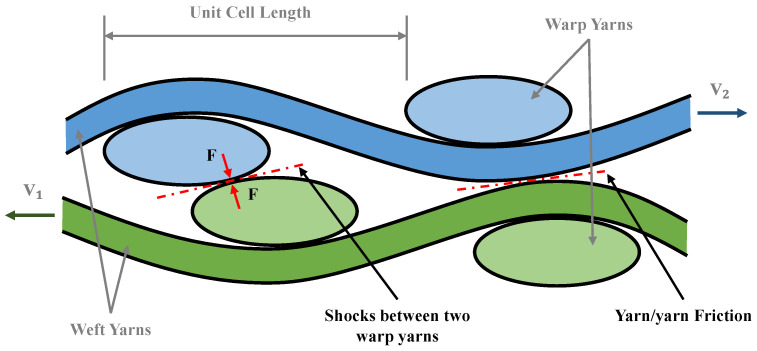
Two different friction behaviors between yarns.

**Figure 22:**
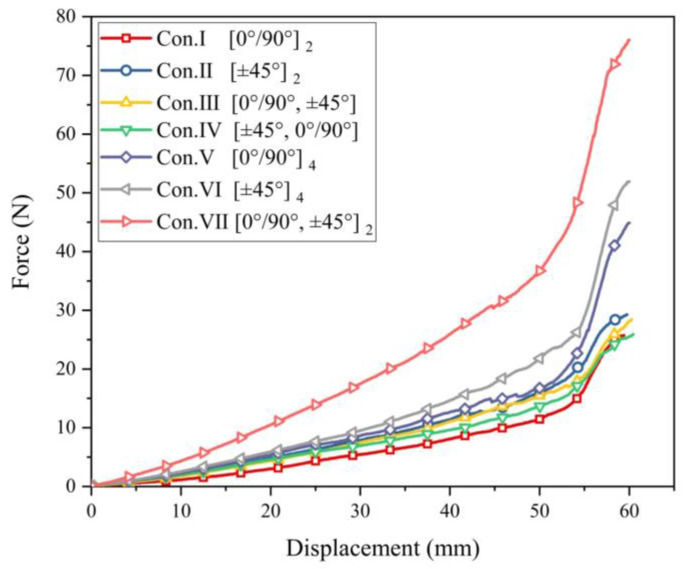
Punch force under different configurations in multi-layer preforming.

In multi-layer preforming, the friction behavior between yarns is the superposition of sliding friction and shock between yarns, as shown in Figure 21. In the current work, the sliding friction between yarns does not cause defects. Another phenomenon is the shock between yarns, especially when out-of-plane deformation occurs. Different plies of yarns would be embedded. When relative slip occurs between plies, these embedded yarns squeeze with each other, resulting in a sharp rise in friction. Compared to the multi-layer configuration with consistent relative orientation between plies, the configuration with inconsistent relative orientation between plies has a more significant relative slip due to the difference in the deformation mode of each ply. Nevertheless, some out-of-plane deformation is inevitable, so the possibility of defects is greater. Figure 22 shows the punch force under different configurations in multi-layer preforming. In the two-layer preforming, the punch force of configuration I is the lowest on the whole, and that of configuration IV is the highest. The punch force of configuration III at some positions even exceeds configuration IV. This proves the rationality of the above inference. Finally, in the four-layer preforming configuration VII, the punch force reaches the highest value. This is obviously not only due to the sliding friction between yarns, but the shock between yarns contributed much friction. Hence, the relative movement of each ply should be considered and reduced as much as possible. This reduction in friction is critical to the quality of the preform. Controlling the shock between yarns in the preforming process is one of the methods to reduce defects. Furthermore, introducing a matted fabric between plies can also reduce friction [46].

## 4. Conclusions

This paper investigated the mechanical behaviors of the carbon-Kevlar hybrid woven reinforcement and its deformation behaviors during the preforming process. The tensile properties of hybrid woven reinforcements exhibited a unique “double peak” phenomenon caused by the different properties of two kinds of fibers. The shear stiffness of the reinforcement was very low, and small wrinkles occurred at the shear angle of about 60°. Neither the tensile nor shear behaviors presented sensitivity to loading rate. In the preforming experiments, wrinkling and yarn breakage were not observed in the useful area of the preform, while significant yarn slippage and out-of-plane yarn buckling were observed in all the preforms. These defects were closely related to the punch shape and the initial yarn orientation and cannot be eliminated by just increasing blank holder pressure. In addition, in multi-layer preforming, the compaction between plies and the friction between yarns simultaneously affect the preform quality. Both factors resulted in a wider range of defects observed in multi-layer preforms than in single-layer preforms, while the locations and defect types were consistent in them. The results obtained in this paper can provide useful guidance for determining the optimized preforming parameters for the hybrid woven reinforcement. In the future, based on the knowledge obtained in this paper, numerical modeling of the preforming process of carbon-Kevlar hybrid woven reinforcement will be conducted to better understand the defect formation mechanism in the preforming.

## Figures and Tables

**Table 1:** Material parameters of carbon-Kevlar hybrid woven reinforcement.

Material	Yarn Count (Yarn/cm^2^)	Woven Structure	Areal Density (g/m^2^)	Thickness (mm)	Yarn Type	Linear Density (g/m)	Bulk Density (g/cm^3^)
Carbon-Kevlar hybrid reinforcement	5 × 5	1/3 twill	220	0.3			
Carbon yarn					T300 3K	0.198	1.76
Kevlar yarn					1500D	0.1679	1.414

## Data Availability

The data presented in this study are available on request from the corresponding author.
